# Development of standardized laboratory methods and quality processes for a phase III study of the RTS, S/AS01 candidate malaria vaccine

**DOI:** 10.1186/1475-2875-10-223

**Published:** 2011-08-04

**Authors:** Christine Swysen, Myriam Bruls, Sunny Oyakhirome, Chris Drakeley, Brenda Okech, Terrell Carter, Adriano Duse, Andrea Reijman, Charlotte Ingram, John Frean, Bernhards Ogutu, the Clinical Trials Partnership Committee

**Affiliations:** 1 GlaxoSmithKline Biologicals, Rixensart, Belgium; 2 Institute of Tropical Medicine, University of Tübingen, Tübingen, Germany; 3 Albert Schweitzer Hospital, Kumasi, Gabon; 4 London School of Hygiene and Tropical Medicine, University of London, London, UK; 5 PATH Malaria Vaccine Initiative, Washington, USA; 6 University of the Witwatersrand Medical School, Johannesburg, South Africa; 7 Contract Laboratory Services, Johannesburg, South Africa; 8 National Health Laboratory Service, National Institute for Communicable Diseases, Johannesburg, South Africa; 9 Malaria Clinical Trials Alliance (MCTA), INDEPTH Network, Accra, Ghana

## Abstract

**Background:**

A pivotal phase III study of the RTS,S/AS01 malaria candidate vaccine is ongoing in several research centres across Africa. The development and establishment of quality systems was a requirement for trial conduct to meet international regulatory standards, as well as providing an important capacity strengthening opportunity for study centres.

**Methods:**

Standardized laboratory methods and quality assurance processes were implemented at each of the study centres, facilitated by funding partners.

**Results:**

A robust protocol for determination of parasite density based on actual blood cell counts was set up in accordance with World Health Organization recommendations. Automated equipment including haematology and biochemistry analyzers were put in place with standard methods for bedside testing of glycaemia, base excess and lactacidaemia. Facilities for X-rays and basic microbiology testing were also provided or upgraded alongside health care infrastructure in some centres. External quality assurance assessment of all major laboratory methods was established and method qualification by each laboratory demonstrated. The resulting capacity strengthening has ensured laboratory evaluations are conducted locally to the high standards required in clinical trials.

**Conclusion:**

Major efforts by study centres, together with support from collaborating parties, have allowed standardized methods and robust quality assurance processes to be put in place for the phase III evaluation of the RTS, S/AS01 malaria candidate vaccine. Extensive training programmes, coupled with continuous commitment from research centre staff, have been the key elements behind the successful implementation of quality processes. It is expected these activities will culminate in healthcare benefits for the subjects and communities participating in these trials.

**Trial registration:**

Clinicaltrials.gov NCT00866619

## Background

A candidate malaria vaccine, RTS,S/AS01, is in late-stage clinical trials in infants and children in sub-Saharan Africa. Earlier studies of the RTS,S/AS vaccine have demonstrated a promising safety profile and efficacy in this population [1-7]. Partners in the clinical development of this vaccine include the manufacturer GlaxoSmithKline (GSK) Biologicals, the Program for Appropriate Technology in Health Malaria Vaccine Initiative (PATH-MVI) as the main funding partner, the Malaria Clinical Trials Alliance (MCTA) as the main capacity strengthening funding partner, and the Clinical Trial Partnership Committee (CTPC), consisting of representatives from the research centres where the trials are being conducted.

Although considerable expertise in biochemistry, haematology, microbiology and malaria-related laboratory methods already existed in some of the participating research centres, there were many centres which had yet to develop these capabilities. Therefore, implementation of quality processes during a pivotal phase III trial presented an important opportunity for capacity strengthening of laboratories across the centres conducting the trials. The extent of capacity strengthening depended on the existing scientific and technical knowledge and laboratory infrastructure at each site prior to trial preparation. Sharing best practice experience across laboratories during the capacity strengthening exercise was valuable.

This paper describes the laboratory methods used in the pivotal phase III study of the candidate RTS, S/AS01 malaria vaccine, and highlights how the efforts of the study investigators and the study centre staff, together with support from collaborating partners, have effectively built laboratory capacity to support the conduct of the trial to good clinical practice (GCP) and good clinical laboratory practice (GCLP) regulatory standards [8] in all the 11 African centres participating in the study.

### Quality assurance: implementation and challenge

The quality assurance (QA) programme is based on the criteria of GCLP from the British Association of Research Quality Assurance (BARQA) and the International Committee on Harmonization guidelines on GCP that cover organization, personnel, facilities, equipment, specimens and reagents, performance and process conduct, standard operating procedures (SOPs), documentation, reporting, archiving, and the QA programme (Table 1). Implementation of the QA process during this large and complex trial has involved the services of Contract Laboratory Services (CLS - Johannesburg, South Africa), a highly experienced company in African projects. Presently CLS plays a key role in monitoring and evaluating participating laboratories, including on-site laboratory appraisals, providing training, facilitating external quality assessment (EQA) and advising on equipment and processes. The EQA programme is summarized in Table 2.

**Table 1:** Quality assurance requirements

	Requirement
**Personnel**	Appropriate qualification, experience, training and competency

**Facilities**	Upgraded or newly built, suitable capacity, secured, restricted access, adequate cleanliness and protection from contamination

**Equipment**	Appropriate design and capacity, calibrated, validated and adequately maintained

**Reagents and specimens**	Adequate labelling and storage

**Process performance**	Adequate study planning, validation of methods, good documentation practice, QC (daily and EQA surveys)

**Documentation**	Full traceability of activities

**SOPs**	Written and authorized for all activities

**Archives**	Restricted access, secured and documented

**Audits **	Internal audits External independent audits

QC: Quality Control; EQA: External Quality Assessment; SOP: Standard Operating Procedure

**Table 2:** Measures in External Quality Assessment

	Measure	Organization	# Samples and frequency	In case of failure
**Parasitology**	Grading of microscopists	NICD	20 samples, 3 times per year	Microscopist excluded from reading study slides for 4 months until re-trained and re-assessed

**Biochemistry**	Pass/Fail^1^	RCPA, I-EQA	1 sample every 2 weeks	1 failure
				• Prepare deviation and action plan
				• Check internal QC
				• Run maintenance check and change reagents and controls
	
**Haematology**	Satisfactory/ Unsatisfactory^2^	I-EQA	2 samples per month	2 failures
				• Prepare deviation and action plan
				• Check internal QC
				• Run maintenance check and recalibrate equipment
				• QA manager to double check QC for failed period
				All cases
				• Interrupt testing on failure equipment (use back-up)
				• If no equipment is operational, discuss possibility of interrupting vaccination

**Bedside testing of glycaemia, base excess, lactacidaemia**	Pass/Fail	Thistle (South Africa)	12 samples 2 times per year	Corrective action implemented

**Microbiology**	Graded 0-4, scores 0 and 1 reflecting low performance	NICD	6 samples 3 times per year	Prepare deviation and action planDiscuss with CLS Retrain staff (by site supervisors and CLS)

NICD: National Institute of Communicable Disease; RCPA: Royal College of Pathology Australasia; I-EQA: International EQA; CLS: Central Laboratory Services (Johannesburg, South Africa); ^1 ^criteria defined by International EQA and RCPA; ^2 ^criteria defined by International EQA

Building infrastructure at some of the centres was an important part of the implementation of the overall QA process. Although some participating centres already possessed state-of-the-art facilities, others lacked some basic requirements such as an adequate power supply. Bringing a level of parity between the centres was, therefore, necessary. Provision of new infrastructure, such as the construction of laboratories and installation and maintenance of equipment, was made possible by financial support from MCTA and resource investments from GSK Biologicals and MVI.

Other practical challenges faced in the development and implementation of the laboratory strengthening program were high staff turnover, with consequent loss of experienced and trained personnel, lack of technical knowledge among equipment vendors, and delays in supply which hampered the installation of equipment. Logistical difficulties included limitations in available transport, compounded by long transportation distances and the challenging climatic conditions, which presented problems in the operation of equipment and preservation of samples and reagents.

### Slide reading for malaria parasites: methodology and quality assurance/quality control

Accurate measurement of the *Plasmodium falciparum *count in blood samples is a vital part of the phase III efficacy evaluation of the RTS,S/AS01 malaria candidate vaccine, as it is crucial for the diagnosis of clinical malaria and severe malaria, the key endpoints for evaluation of vaccine efficacy. Different methods exist for counting parasite density; however, the World Health Organization (WHO) expert panel on the measurement of malaria vaccine efficacy in phase III clinical trials recommends a method based on reading linked to a defined number of white blood cells and use of individually calculated white cell counts [9].

The methods used for the trial were established by a CTPC working group, consisting of investigators, representatives of GSK Biologicals and MVI, as well as external experts from CLS and the South African National Institute for Communicable Diseases (NICD). The working group assessed different available methods for reproducibility, ease of use, and accuracy, and agreed on two methods as acceptable. Method 1 counts *P. falciparum *parasites against a known white blood cell concentration determined at the same time as the smear is performed, and follows the principles described by Greenwood and Armstrong [10]. Method 2 counts against an assumed known blood volume and follows the principles described by Planche *et al *[11]. The consistency of results from the two methods is established by performance assessment.

### Parasite density counting methodology

Blood samples are collected by venipuncture or finger or heel prick and either transferred to the slide directly or to a microcontainer containing ethylene diamine tetraacetic acid (EDTA). Two slides per subject are prepared. The asexual blood stage parasites of all parasite species are identified at 100× magnification.

#### Method 1: counting against known blood cell concentration

**Figure 1:**
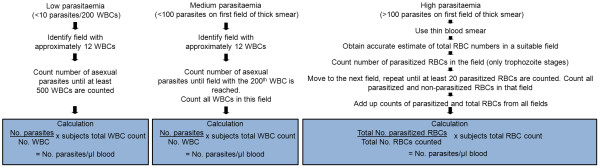
**Parasite density counting methodology (method 1)**.

One thin and one thick smear per slide are made, using 2 μL of blood for the thin smear and 6 μL of blood for the thick smear using a template. Slides are stained using Giemsa. A graphic representation of the methodology is shown in Figure 1. A minimum of 100 fields needs to be counted on the thick smear before a slide is recorded as negative. If the parasite density is less than 10 per 200 white blood cells on the thick smear, the number of parasites per 500 white blood cells is counted. If the parasite density is more than 10 but less than 100 in the first field of the thick smear, the number of parasites is counted per 200 white blood cells. If 100 or more parasites are seen in the first field of the thick smear, the parasite density is assessed on the thin blood smear as the proportion of parasitized red blood cells in the total number of red blood cells counted. The red blood cells and parasitized red blood cells are counted on the thin blood smear until a minimum of 20 parasitized blood cells is counted. If 20 parasitized red blood cells are counted before all the red blood cells in the field are counted, counting is finished in that field. The subject's true blood cell count is used in the calculation of the number of parasites per microlitre of blood; complete blood counts are performed immediately after collection using an automated method.

#### Method 2: counting against known blood volume

In this method, 10 μL of blood is spread onto an area of 1 × 1.8 cm, using a template. Slides are stained using Giemsa. One hundred fields must be parasite-free for a slide to be declared negative. If there are between one and nine parasites per field, 100 fields are counted; if there are 10-99 parasites per field, 10 fields are counted; if there are 100-999 parasites per field, one field is counted. The parasite count is calculated according to the formula: parasites/μL = parasites/field x microscope factor. When >1 field is counted, the mean number of parasites/field is calculated. The microscope factor - predetermined for each microscope - is the assumed blood volume per microscope high power field. To calculate the microscope factor, the number of white blood cells is counted across 10 fields, and the mean number per field is calculated. The number of white blood cells in 10 μL of blood is counted immediately after collection using an automated blood analyzer. The microscope factor is equal to the number of white blood cells in 10 μL of blood (automated analyzer) divided by the mean number of white blood cells per high power field.

#### Criteria for concordance and determination of final result

All slides are read twice by two independent microscopists. A third independent microscopist also reads the slide if there are any of the following discrepancies between the first two readings: (1) a positive reading by one microscopist and a negative reading by the other; (2) both microscopists record a parasitaemia >400 parasites/μL but the higher count divided by the lower count is >2; (3) at least one microscopist records a parasitaemia ≤400 parasites/μL but the higher reading is more than 10 times the lower reading.

**Figure 2:**
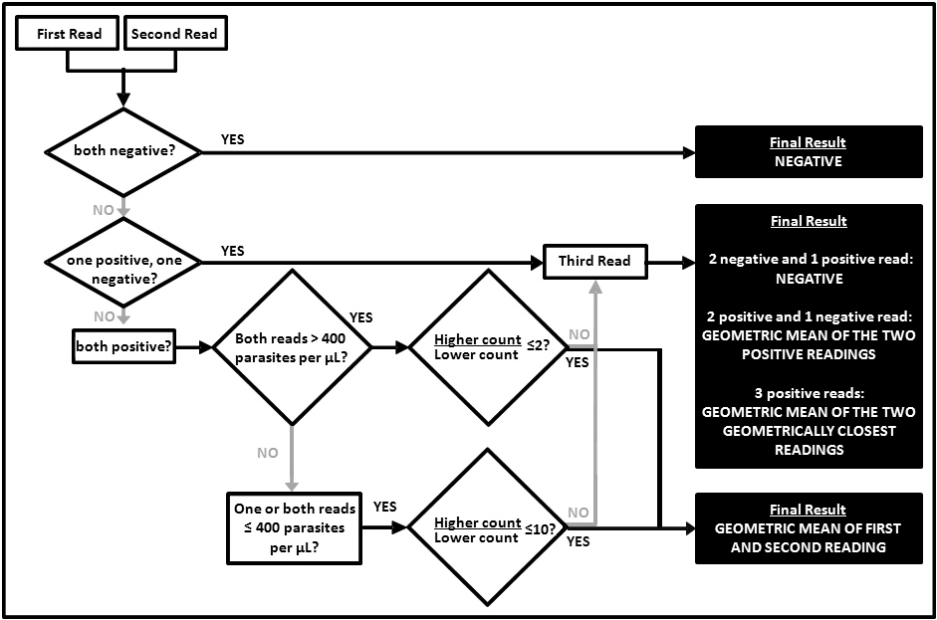
**Parasite density counting: criteria for concordance of readings and determination of final result**.

If the initial two readings give concordant results, the final parasite density is considered to be the geometric mean of the two readings. If the readings are discordant, then the following principles are applied: (1) where one reading is positive and the other negative, the majority decision obtained following the reading by the third microscopist is adopted - if positive, the final result is the geometric mean of the two positive results; (2) where all three readings are positive, the final result is the geometric mean of the two closest readings (Figure 2).

### Slide reading performance measurement

After each preparation of a new batch of stain, internal quality control (QC) is performed on one negative and one positive slide. The external QA process for slide reading comprises species identification and parasite quantification. It is based on WHO recommendations [12] (Table 3).

**Table 3:** Grading of competency in parasite microscopy (species identification, and quantification)

Grading*	Species identification (% accuracy)	Quantification (% slides within 25% of true count)
Expert	90	50

Reference	80	40

Competent	70	30

In training (failure)	<70	<30

*Grading according to WHO criteria [12]

Three assessments per year are planned and include 20 samples per microscopist. The samples in each assessment batch are provided by NICD (South Africa) and present a variety of challenges, e.g. negative samples, samples with very high counts, samples repeated to evaluate consistency (within the current survey and from previous surveys), samples with non-falciparum species and non-malaria pathogens. The true value of each sample parasite count is taken to be the median of the values obtained from the Parasitology Reference Unit of the NICD, two WHO reference laboratories and the laboratories of the study centres. Microscopists who fall below the level defined as competent are considered to be 'in training' and are not allowed to read study slides until retrained and re-assessed (Table 3).

### Biochemistry and haematology: methodology and quality assurance/quality control

Before participating in the trial, many sites used manual methods for biochemical and haematological tests. However, it was decided to introduce automated equipment for these tests in order to improve reliability, increase sample handling per day, reduce contamination risk, reduce blood volume requirement and enhance QA/QC assessment. Equipment for bedside measurements (blood gases, glucose and base excess), and other biochemical and haematological assays was chosen on the basis of performance, capacity, ease of use and availability of local technical support. The choice of equipment was made by individual centres, in collaboration with CLS. Duplicates of each major piece of equipment (main and back-up) were supplied. Two trained personnel operate each piece of equipment. Significant efforts by study centre personnel were required in order to overcome the many challenges faced during the installation of equipment, including delivery logistics and accessing reagents. Also, qualifying the methods before study start on the respective equipment proved a long and difficult exercise due to lack of prior experience and availability of samples.

All biochemistry automated analyzers were initially enrolled with International EQA, formerly known as UKNEQAS (UK National External Quality Assessment Service) and then switched to the Royal College of Pathologists of Australasia (RCPA), because the latter was more appropriate for the study requirements at the time. All haematology automated analyzers are enrolled in EQA by UKNEQAS. Critical equipment such as analyzers, autoclaves, fridges/freezers, incubators, laminar flow hoods and bedside equipment (I-STAT and Nova) were validated at installation by the vendor, who checked performance parameters and ran a calibration as needed. After this, a detailed installation qualification-operational qualification (IQ-OQ) certificate was provided. The users then performed some tests and ran QC samples to check the performance under routine conditions, reporting it in a performance quality (PQ) document. Thereafter, a yearly calibration is run by the vendors. For every piece of equipment, all interventions and incidents are recorded in a logbook. Each laboratory has needed to demonstrate method qualification for biochemistry and haematology. This includes analysis of repeatability and reproducibility on 10 samples covering the analysis range that is tested in duplicate on two to three independent runs. In addition, it includes analysis of linearity, tested by consecutive dilutions of five samples, and accuracy or bridging of a panel of 60 samples between main and back-up analyzing equipment. For the latter, very low titered bilirubin samples are avoided, because bridging was proved to be difficult in the low ranges, while low ranges are of no concern for safety. Finally, stability of the QC samples is documented in the method qualification reports.

To show stability of the equipment, two or three QC samples are tested daily until the expiration date of the batch, and Levy-Jennings charts [13] of the test results are created to monitor whether they remain within the acceptable limits as provided by the supplier of the QC samples. One biochemistry QC sample (for bilirubin, creatinine and alanine aminotransferase) is sent to each laboratory every two weeks, and the criteria of acceptability are defined by International EQA and the RCPA (results are pass or fail). Two haematology samples (for white blood cell count, red blood cell count, platelet count, and haemoglobin concentration) are sent to each laboratory every month; acceptability criteria are defined by International EQA (results are satisfactory or unsatisfactory). QC samples for EQA of bedside equipment to be analyzed over the subsequent six months are sent to each laboratory twice yearly by Thistle QA, South Africa (Table 2). Performance measurements by EQA are shown in Table 2. It is important to note that performance was better when QC reagents are provided by the vendor of the equipment.

### Microbiology: methodology and quality assurance/quality control

Microbiological testing is essential for the diagnosis of malaria and associated co-morbidities. The primary case definition of severe malaria, adopted for evaluation of endpoints in this phase III trial, requires the exclusion of several co-morbidities including sepsis and meningitis, which are defined by a positive blood or cerebrospinal fluid culture [14].

Microbiology facilities in many participating centres had to be set up from the beginning, including installation of dedicated laboratories, purchase of equipment, and staff training. A working group consisting of expert consultants and representatives from collaborating parties recommended standard microbiology methods using automated Bactec™ incubators and paediatric bottles (Bactec BD Diagnostic Systems, USA). The considerations that led to the choice of the automated system were standardization of methodology across sites, the high sensitivity of the Bactec™ system with reduced chances of contamination, and the availability of technical support. Conventional systems are labour intensive and require skilled operators. This is an important consideration when skilled people are scarce and turnover is high.

Positive blood cultures are detected by carbon dioxide production, and positive automated cultures are sub-cultured using standard methods [15,16]. Further bacterial identification relies on the API bioMerieux™ and BBL CRYSTAL™ methods supported by computerized systems that generate the name of the most likely organism based on the biochemical reactions obtained. Additional manual rapid tests used to differentiate between organisms and/or groups of organisms include oxidase, catalase and indole biochemical testing. Serotyping is used for identification of Salmonella and Shigella species. Rapid latex identification kits are used for confirmation of staphylococci, streptococci and pneumococci.

For the purpose of the trial analysis, as opposed to clinical care, there was an effort to standardize pathogen classification, according to a previously published algorithm [16] that was endorsed by a microbiology working group composed of representatives of the investigator groups and international experts in microbiology in Africa. A blood culture is considered positive if a definite pathogen is isolated (e.g. *Streptococcus pneumoniae*, *Streptococcus agalactiae*, *Streptococcus pyogenes*, *Haemophilus influenzae*, *Salmonella *species) or if a bacterium that could be either a pathogen or a contaminant is isolated within 48 hours of incubation (e.g. *Escherichia coli*, *Klebsiella pneumoniae*, *Staphylococcus aureus*, *Enterococcus faecalis*). A blood culture is considered to be contaminated if a likely contaminant is isolated or if a bacterium that could be either a pathogen or a contaminant is isolated after more than 48 hours of incubation.

CSF is collected by lumbar puncture, biochemical (glucose and protein) analyses and a white blood cell count is performed. Gram-stained direct smears are examined microscopically for the presence of bacteria, CSF is cultured using internationally accepted standardized microbiological methods, and bacterial growth is checked for after incubation for 24 and 48 hours. Bacterial identification is performed according to methods described previously, as well as latex antigen agglutination tests for *S. pneumoniae*, *H. influenza *and *Neisseria meningitidis*.

### Microbiology performance measurement

Microbiology EQA covers evaluation of microscopy, culture and identification, serotyping, choice of antimicrobial for particular isolates, and antimicrobial susceptibility testing (Table 2). Six samples (with at least two meningeal and two enteric organisms) are sent to each laboratory three times per year as part of the NICD microbiology EQA, and the criteria of acceptability are defined by NICD (Table 2). Internal quality control using American Type Culture Collection control strains for species identification testing is performed weekly, when a new batch of reagent is received or when discordant results occur. Internal QC is carried out on all antimicrobial discs available for antimicrobial susceptibility testing for use in the lab. The contamination rate of the clinical specimens is evaluated monthly by internal assessment. Continuous assessment allows re-training programmes for both hospital and laboratory staff and more intense quality evaluation in case of high contamination rates.

## Conclusions

Development of standardized laboratory methods and quality assurance and control processes in the ongoing phase III study of the RTS,S/AS01 candidate malaria vaccine is an example of a successful collaboration between research centres, trial sponsors and funding partners. Despite major challenges, study centres made tremendous achievements in leading this process, along with the sponsor and funding partners who provided support, training and coordination.

The focus has been to develop and maintain standardized laboratory facilities and procedures that meet current criteria for the conduct of clinical trials. High importance has been given to QC and QA in this study. External QA assessment of all laboratory methods (haematology, biochemistry, parasitology, microbiology) and bedside measurements are key aspects of this process. Automated equipment has replaced manual methods previously used by some centres. Through implementation of the QA systems and standard operation procedures covering all activities, the study centres have been able to effect considerable improvements in staff knowledge and competency.

Whilst this paper demonstrates what is possible, there remain challenges for the sustainability of the developed capacity described in this paper [17-19]. Although only equipment that could be serviced in country was selected, expensive consumables and maintenance contracts will be required after the trial. Many staff have been trained and assessed continuously in laboratory techniques and these capable and experienced staff must be retained. However, the research centres now have greater capabilities and the potential to diversify their research portfolios beyond malaria. A number of centres have already leveraged this capacity to successfully attract research grants. Given the limited microbiology services in some areas, particularly rural areas, another opportunity that is being explored by some centres is to act as a national reference laboratory for microbiology or as a site for sentinel surveillance. All centres together with MCTA are looking at ways for the capacity to be sustained and to contribute to research and health care provision.

In conclusion, huge progress has been made in putting standardized methods into place and developing QA/QC processes in various research laboratories in Africa. There is adequate capacity to perform laboratory evaluations required in the conduct of the pivotal phase III study of the RTS,S/AS01 paediatric malaria candidate vaccine. Therefore, partners in the clinical development of this advanced malaria vaccine candidate are confident that the laboratory evaluations can be performed to the high standards required for clinical trials. Crucially, the capacity strengthening now in place will ensure a positive, significant effect on the potential to conduct other research and healthcare benefits for the communities participating in this trial on its conclusion.

## Competing interests

CS, JV, MB, OOA, and BOk are employees of GSK Biologicals or were so during the writing of this manuscript. JV holds stock options in GSK Biologicals. CS, JV, MB, PK, BG, OOA, Bok, AR, CI, and JF declare their institution received a grant from MVI for the clinical trial described in this manuscript. JV, MB, and OOA declare their institution has received grants from MVI for previous clinical trials. BG, AD, AR, CI, and JF declare receiving travel funds from GSK Biologicals for travel related to this clinical trial. AR and CI declare their institution receiving financial support for lab support from GSK Biologicals. During the development of this manuscript, TV, TC, and BS were employed by MVI. SO and BOg declare no potential conflicts of interest.

## Authors' contributions

All authors contributed to the development of this manuscript through discussions and document review. CS led the writing of the manuscript coordinating the incorporation of all reviewer comments. JV and BS coordinated the input of the MVI team. All authors critically contributed to the discussions on laboratory methodology. All authors read and approved the final manuscript.
